# Engineering of Recombinant Poplar Deoxy-D-Xylulose-5-Phosphate Synthase (*Pt*DXS) by Site-Directed Mutagenesis Improves Its Activity

**DOI:** 10.1371/journal.pone.0161534

**Published:** 2016-08-22

**Authors:** Aparajita Banerjee, Alyssa L. Preiser, Thomas D. Sharkey

**Affiliations:** Department of Biochemistry and Molecular Biology, Michigan State University, East Lansing, MI, United States of America; Russian Academy of Medical Sciences, RUSSIAN FEDERATION

## Abstract

Deoxyxylulose 5-phosphate synthase (DXS), a thiamine diphosphate (ThDP) dependent enzyme, plays a regulatory role in the methylerythritol 4-phosphate (MEP) pathway. Isopentenyl diphosphate (IDP) and dimethylallyl diphosphate (DMADP), the end products of this pathway, inhibit DXS by competing with ThDP. Feedback inhibition of DXS by IDP and DMADP constitutes a significant metabolic regulation of this pathway. The aim of this work was to experimentally test the effect of key residues of recombinant poplar DXS (*Pt*DXS) in binding both ThDP and IDP. This work also described the engineering of *Pt*DXS to improve the enzymatic activity by reducing its inhibition by IDP and DMADP. We have designed and tested modifications of *Pt*DXS in an attempt to reduce inhibition by IDP. This could possibly be valuable by removing a feedback that limits the usefulness of the MEP pathway in biotechnological applications. Both ThDP and IDP use similar interactions for binding at the active site of the enzyme, however, ThDP being a larger molecule has more anchoring sites at the active site of the enzyme as compared to the inhibitors. A predicted enzyme structure was examined to find ligand-enzyme interactions, which are relatively more important for inhibitor-enzyme binding than ThDP-enzyme binding, followed by their modifications so that the binding of the inhibitors can be selectively affected compared to ThDP. Two alanine residues important for binding ThDP and the inhibitors were mutated to glycine. In two of the cases, both the IDP inhibition and the overall activity were increased. In another case, both the IDP inhibition and the overall activity were reduced. This provides proof of concept that it is possible to reduce the feedback from IDP on DXS activity.

## Introduction

The 2-methyl-3-erythritol-4-phosphate (MEP) pathway is one of the biochemical pathways that lead to the biosynthesis of isopentenyl diphosphate (IDP) and dimethylallyl diphosphate (DMADP), the building blocks of isoprenoids [1–5]. Isoprenoids constitute a diverse group of natural products occurring in all different forms of life including animals, plants as well as bacteria and archea. Many isoprenoids have important commercial applications as drugs, natural polymers, pigments, flavor and fragrance molecules, agrichemicals, cosmetics, biofuels etc. [6–11]. Natural sources of some isoprenoids is limited [7, 9, 12], therefore, the biotechnological production of commercially important isoprenoids has attracted widespread attention as a valuable industrial target [11].

Metabolic engineering of the MEP pathway has become a focus to improve the bio-production of downstream isoprenoids [13]. 1-Deoxy-D-xylulose-5-phosphate synthase (DXS), the first enzyme of the MEP pathway, has been one of the main targets for engineering. Over-expression of exogenous DXS from prokaryotic origin has been found to increase the biosynthesis of carotenoids and ubiquinone in *E*. *coli* [14]. It has also been shown that coexpression of DXS with other downstream enzymes improved the yield of taxadiene, lycopene, sesquiterpenes, various mono and diterpenes, carotenoids including β-carotene, and zeaxanthin in *E*. *coli* [15–21].

The reaction mechanism of DXS involves binding of ThDP at the active site of the enzyme, formation of a covalent intermediate between enzyme-bound ThDP and pyruvate, followed by glyceraldehyde-3-phosphate (GAP)-stimulated decarboxylation of the bound pyruvate, then addition of GAP to the remaining two carbon fragment to make 1-deoxy-D-xylulose-5-phosphate (DXP) [22]. A recent study demonstrated that DXS is inhibited by both IDP and DMADP resulting in feedback regulation [23, 24]. These isoprenoid precursors compete with ThDP for binding at the active site of the enzyme [23]. Competition of ThDP binding at the active site of the enzyme with that of IDP and DMADP indicates that the ThDP is free to dissociate from the enzyme between catalytic events. Therefore, for DXS kinetics, ThDP can be considered as a substrate rather than just being a cofactor. The importance of DXS in the metabolic regulation of the MEP pathway makes it a critical target for engineering so that the manipulated enzyme can overcome the regulatory limitation to achieve an improved MEP pathway for industrial use.

The structural model of DXS from *Populus trichocarpa* (Torr. & A.Gray) (*Pt*DXS) in our previous work predicted the importance of some key residues at the active site of the enzyme in binding ThDP and IDP/DMADP [23]. Both ThDP and the inhibitors share similar binding interactions with *Pt*DXS. However, ThDP being a larger molecule compared to IDP/DMADP, has more anchoring sites for binding with the enzyme. In this study, we tested the effect of two key Ala residues in binding ThDP and IDP and also tested whether the engineering of *Pt*DXS can preferentially reduce the binding affinity of IDP relative to ThDP binding. We chose IDP for examining the inhibition properties of the mutants in this study as both IDP and DMADP exhibit similar behaviors for inhibiting WT*Pt*DXS [23]. Ala-147 and Ala-352 at the active site were selected for mutation as they were critical for binding the carbon chain of IDP and DMADP. These residues were either individually or simultaneously mutated to Gly to generate A147G*Pt*DXS, A352G*Pt*DXS and A147G/A352G*Pt*DXS. A147G*Pt*DXS was found to have improved activity and slightly higher inhibition by IDP compared to the WT. A352G*Pt*DXS also exhibited slightly higher activity and stronger inhibition by IDP compared to the WT. On the other hand, the double mutant was found to have slightly reduced activity and reduced inhibition by IDP compared to the WT. The engineering of A147G/A352G*Pt*DXS could possibly be useful in partially or completely relieving the feedback regulation of the MEP pathway.

## Materials and Methods

### Site-directed mutagenesis

The DXS cloned from *Populus trichocarpa* (Torr. & A.Gray) [23] was the source of the enzyme used in this study. Two sites were chosen for PCR-based site-directed mutagenesis. For A147G*Pt*DXS and A352G*Pt*DXS, the plasmid construct for C-terminally His-tagged WT*Pt*DXS (pET17b3’HR/*Pt*DXS) was used as template. The primers used for A147G*Pt*DXS mutation were 5'-CCT GCT GTC ATA CCT CCA TCA CC-3’ and 5'-GGT GAT GGA GGT ATG ACA GCA GG-3'. The primers used for A352G*Pt*DXS mutation were 5’-CCT CCT CCC ATA CCA GCA TGA ATT GC-3’ and 5’-GCA ATT CAT GCT GGT ATG GGA GGA GG-3’. For the double mutant, PCR-based site-directed mutagenesis was done using the plasmid construct of A352G*Pt*DXS as the template and the primer pairs for A147G*Pt*DXS as primers. The PCR reaction mixture was then subjected to DpnI digestion. PCR-clean up of the reaction mixture from DpnI digestion was done using Promega PCR-clean up kit. The resulting plasmids for different mutants were initially transformed in *E*. *coli* strain DH5α to verify the sequence of the mutants. The presence of appropriate sequence of the mutants and the absence of any undesired mutation were confirmed by DNA-sequencing.

### Overexpression and purification of WT and different mutant *Pt*DXS enzymes

The WT and different mutants of *Pt*DXS were over-expressed and purified following the reported procedure [23] with some modifications. The plasmid constructs for WT and the various mutants of *Pt*DXS were over-expressed in *E*. *coli* strain BL21(DE3)pLysS. In each case, cells were grown, induced, and harvested as reported before [23]. The cells were lysed on ice by sonication (Qsonica sonicator ultrasonic processor, Part No. Q500, Misonix sonicator). The sonicator was set to an amplitude of 35%. Sonication was then carried out with 30 s cycles for 5 min where, each cycle consists of pulses with 15 s ON and 15 s OFF. EDTA-free protease inhibitor cocktail (Sigma, catalog number S8830) was added to the cell suspension (final concentration of ~1X the concentration recommended by the manufacturer) right before sonication. The crude lysate was subjected to ammonium sulfate precipitation followed by Ni-NTA column purification as described before [23]. In the elution step of Ni-NTA column purification, most of the protein was eluted with 100–150 mM of imidazole. A minor difference in procedure for Ni-NTA column purification was used for A147G*Pt*DXS. For this mutant, the column was very slow during the washing step with the wash buffer. A gradient elution buffer could not be used for this mutant and the protein was eluted with the elution buffer containing 250 mM imidazole. After the Ni-NTA column purification, a final ammonium sulfate precipitation, followed by dialysis and storage of the purified protein were done as described before [23].

### Kinetic characterization by LC-MS/MS-based assay

The kinetic analysis of the purified WT and various *Pt*DXS mutants were performed using LC-MS/MS based assay as reported before [23]. Briefly, the assay mixture was prepared by adding 10 mM MgCl_2_, 1mM dithiothreitol (DTT), appropriate concentration of ThDP, 1 unit/ml rabbit muscle triosephosphate isomerase, and 0.25 μM WT/mutant *Pt*DXS to 40 mM Tris-HCl buffer at pH 8.0 in a total volume of 100 μl. The reaction was initiated by adding a mixture of appropriate concentration of dihydroxyacetone phosphate (DHAP) and pyruvate and carried out at 37°C for 5 min. The substrate, GAP, was replaced with a mixture of dihydroxyacetone phosphate (DHAP) and triose-phosphate isomerase from rabbit muscle to maintain its constant supply in the reaction mixture. The equilibrium ratio of DHAP:GAP was calculated to be 18:1 at 37°C [25]. The reaction was then terminated by freezing in liquid nitrogen followed by the addition of 400 μl of ice-cold acetonitrile, keeping the frozen reaction mixture on dry ice. The assay mixture was then thawed on ice and supplemented with 2 μM [^13^C_2_]DXP as an internal standard for the mass spectrometry. Finally, the assay mixture was centrifuged at 28,000 X g for 10 min, and the supernatant was stored at -80°C until further analysis. The amount of DXP produced in each case was then quantified using LC-MS/MS based technique as described previously [23]. Typically, in order to determine the *K*_m_ of a particular substrate, a range of concentration was used for that substrate keeping the concentration of the other substrates constant. Table 1 shows the concentration of various substrates used for the kinetic studies of different variants of *Pt*DXS. The *K*_m_ values of various *Pt*DXS enzymes for different substrates were obtained by fitting the experimental data with non-linear regression using Michaelis-Menten equation in Origin (OriginLab, Northampton, MA). S3 Fig shows the individual Michaelis-Menten plot for each of the enzyme variants for each of the substrates.

**Table 1:** Concentration of different substrates used for the determination of *K*_m_ for various substrates. Rabbit muscle triose phosphate isomerase (1 U/mL) was added and an equilibrium ratio of 18:1 for DHAP:GAP was used to assign a concentration of GAP.

Substrate tested (for determination of *K*_m_)	Concentration of substrates used in each reaction		
	ThDP (mM)	DHAP (μM)	Pyruvate (mM)
ThDP	0‒1	263	5
GAP	0.1	0–197	5
Pyruvate	0.1	263	0–1

### Inhibition studies

IDP was purchased from Isoprenoids, LC (Tampa, FL, USA). The inhibition assay for each of the enzymes was carried out as described before in the presence of ~*K*_m_ concentration of ThDP and ~2 X *K*_m_ concentration of DHAP and pyruvate [23]. The concentration of IDP used in the inhibition assay was 0–1 mM. The non-linear fitting of the IC_50_ curves was done as described before using the program Origin (OriginLab, Northampton, MA) [23]. The determination of *K*_i_ of each of the enzymes for IDP was done from the IC_50_ curves as reported earlier [23] by fitting the equation

$$K_{\text{i}}=\;\frac{IC_{50}}{1+\;\frac{\left[S\right]}{K_{\text{m}}}}.$$

## Results

### Site-directed mutation, overexpression, and purification of *Pt*DXS

Computational modeling studies predicted that both ThDP and IDP use similar polar interactions for binding of the diphosphate moiety with the enzyme and pyrophosphate was not an inhibitor of DXS [23]. Therefore, any mutations involving these polar interactions may affect the binding of ThDP as well as IDP and would be unlikely to improve the kinetics of the enzyme. To select interactions essential for IDP binding, but not as important for the binding of ThDP we focused on those residues of the enzyme that are important for binding the carbon chain of IDP, reasoning that the diphosphate binding was likely to be similar between ThDP and IDP.

**Fig 1:**
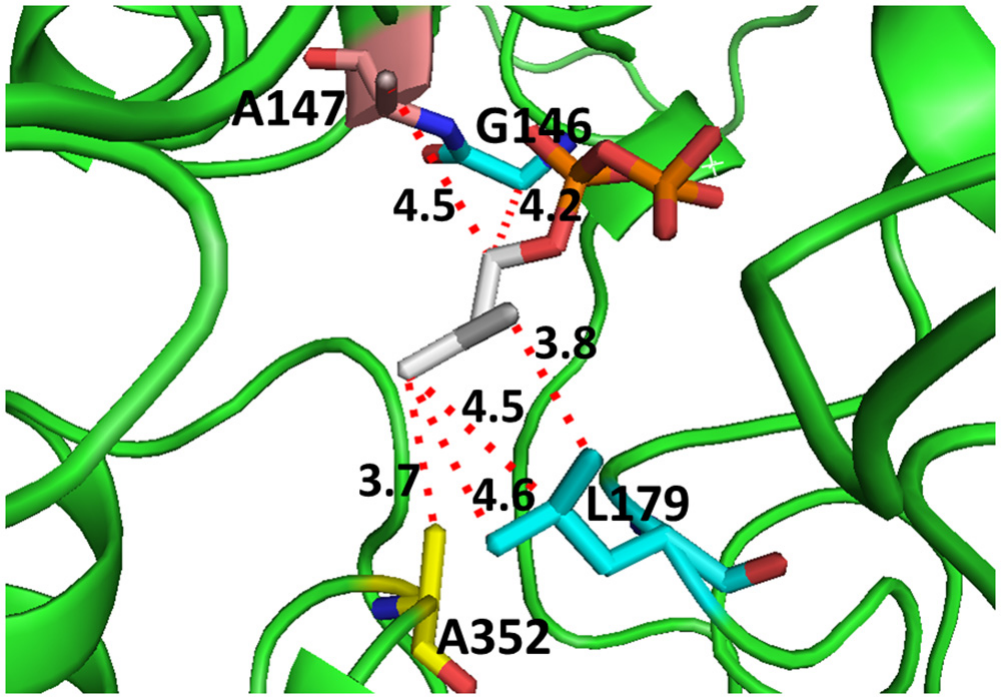
Cartoon view of the interactions of different residues of WT*Pt*DXS with IDP and their relevant distances from the carbon chain of IDP.

The computational modeling study predicted that the carbon chain of IDP would have non-polar interactions with Leu-179, Ala-352, Gly-146, and Ala-147 (Fig 1). These interactions of the carbon chain of IDP are important in binding this molecule at the active site of the enzyme. Among these residues, Gly-146 is also involved in the polar interaction with the diphosphate group [23] and Leu-179 has several nonpolar interactions with the C2 atom and the sulfur atom of the thiazolium ring of ThDP (S1 Fig). Computational modeling studies in our previous work [23] also predicted that the methyl side chain of both Ala-147 and Ala-352 residues has van der Waals interactions with both the terminal carbon atoms of IDP and thus provide anchoring sites for the beginning and the end of the carbon chain of IDP. Therefore, mutations involving these alanine residues to glycine might affect these van der Waals interactions resulting in poor binding of IDP with the enzyme. On the other hand, ThDP potentially has numerous other important hydrophobic interactions at the active site of the enzyme to compensate the loss of its interactions with these Ala residues. These mutations, therefore, could lead to selective binding of ThDP over IDP. Therefore, Ala-147 and Ala-352 were selected for mutation to glycine to selectively reduce binding of IDP more than that of ThDP. Site-directed mutagenesis was carried out to generate A147G*Pt*DXS, A352G*Pt*DXS, and the double mutant A147G/A352G*Pt*DXS. The purified mutant proteins have similar molecular weight as expected and similar expression level in *E*. *coli* as that of the WT as observed by SDS-PAGE (S2 Fig).

### Kinetics of different mutants of *Pt*DXS

**Fig 2:**
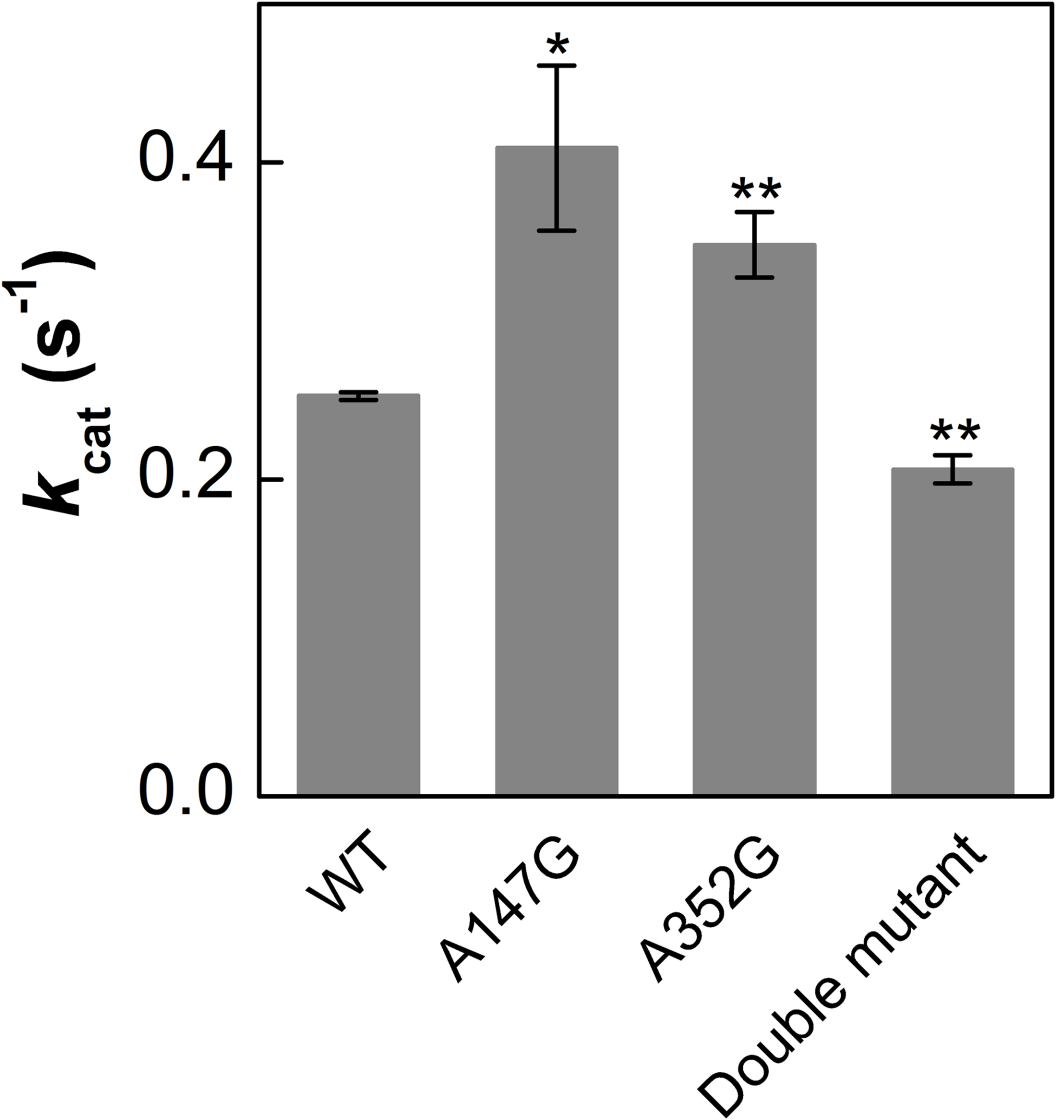
Comparison of the *k*_cat_ of WT and various mutants of *Pt*DXS based on LC-MS/MS based assay. Each bar represents mean, error bars represent S.E. (n = 3). A147G*Pt*DXS showed highest turnover number among all of them. Data with an asterisk (*) are significantly different from WT as determined by Student’s *t*-test. (*P < 0.05 and **P ≤ 0.01).

The kinetic parameters of the different mutants of *Pt*DXS were determined and compared with that of the WT. Table 2 and Fig 2 show the *K*_m_ and *k*_cat_ values of the WT and the various mutants of *Pt*DXS for different substrates. The *K*_m_ values of the mutants were higher for ThDP than that of WT (~ 2.8, ~2.9, and ~10.9-fold higher for A147G*Pt*DXS, A352G*Pt*DXS, and A147G/A352G*Pt*DXS respectively). No inhibition was observed even at the highest concentration of ThDP despite it being extremely high relative to the *K*_m_ (S3 Fig). For pyruvate, the *K*_m_ values of the mutants were higher than that of WT (~ 1.9, ~2, ~2.6-fold higher for A147G*Pt*DXS, A352G*Pt*DXS, and A147G/A352G*Pt*DXS respectively).The mutants had comparable *K*_m_ values for GAP as that of the WT (Table 2). The *k*_cat_ of A147G*Pt*DXS (~ 0.4 s^-1^) is higher than that of WT. A352G*Pt*DXS had a slightly higher *k*_cat_ and A147G/A352G*Pt*DXS had a slightly lower *k*_cat_ than the WT (Fig 2).

**Table 2:** Kinetic constants and inhibition constant (*K*_*i*_) of the WT and various mutants of *Pt*DXS measured by LC-MS/MS-based assay. For *K*_m_, each number represents mean ± S. E. (n = 3). For *K*_*i*_, each number represents the calculated value from the non-linear fitting of the IC_50_ curve ± S. E. (n = 3).

*Pt*DXS	*K*_m_ (μM)			Catalytic Eff. (ThDP) *k*_cat_/*K*_m_ (M^-1^ s^-1^)	*K*_*i*_ (μM)
	ThDP	Pyruvate	GAP		IDP
WT	8 ± 3	105 ± 63	5 ± 1	31587	92 ± 8
A147G	22 ± 12	201 ± 47	5 ± 1	18599	73 ± 2
A352G	23 ± 12	208 ± 45	7 ± 1	15217	31 ± 4
A147G/A352G	87 ± 13	268 ± 71	5 ± 2	2372	268 ± 75

### Inhibitory effect of IDP on different mutants of *Pt*DXS

**Fig 3:**
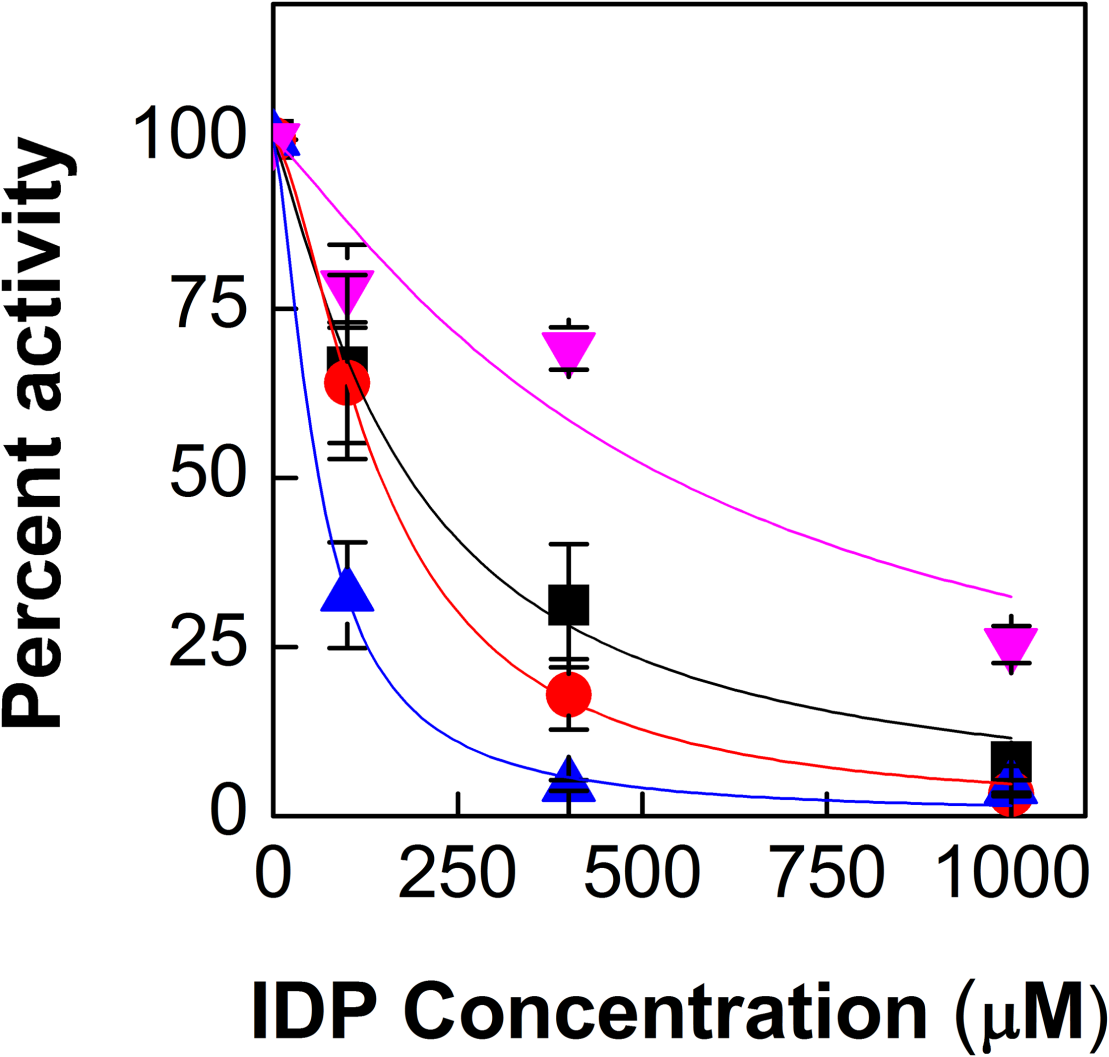
Effect of IDP on WT and different mutants of *Pt*DXS. Each data point represents mean, error bars represent S.E. (n = 3). Different symbols represent the experimental data points. The solid lines represent the fitted IC_50_ curves. Black, red, blue, and pink represent the activity of WT, A147G, A352G and A147G/A352G*Pt*DXS respectively. A147G*Pt*DXS/A352G*Pt*DXS showed least inhibition by IDP.

Fig 3 shows the activity of different mutants of *Pt*DXS over a broad range of concentration of IDP in the presence of *K*_m_ concentration of ThDP. The *K*_*i*_ values of IDP were calculated from the non-linear fitting of the IC_50_ curve [23] and are shown in Table 2. Fig 3 shows that A147G*Pt*DXS and A352G*Pt*DXS are much more sensitive to IDP as compared to the WT. The *K*_*i*_ values of IDP for A147G*Pt*DXS was found to be 73 ± 2 μM and this value is ~ 0.8 times the corresponding values for the WT. A352G*Pt*DXS was also found to have stronger inhibition than the WT (Fig 3) with the *K*_*i*_ values of 31 ± 4 μM for IDP which is ~0.3 times that of the WT. On the contrary, the double mutant A147G/A352G*Pt*DXS was also shown to have less inhibition by IDP as compared to the WT (Fig 3). For this mutant, the *K*_*i*_ value of IDP was found to be 268 ± 75 μM which is ~2.9 times higher than that of the corresponding values for the WT.

## Discussion

Modifications of the active site increased the *k*_cat_ of DXS, but only the double mutant reduced binding of IDP. However, the *K*_m_ for ThDP was also increased and so binding of the inhibitor was not preferentially reduced relative to ThDP. The catalytic efficiency (*k*_cat_/*K*_m_) of all the mutants was lower than that of the WT. This resulted from greater increase in *K*_m_ relative to *k*_cat_ (Table 2). The higher *k*_cat_ of the single mutants make them suitable for biotechnological applications and the reduced sensitivity of the double mutant to IDP likely would allow a higher flux through the MEP pathway in the presence of a higher concentration of IDP, which could aid downstream reactions. The higher the ThDP concentration, the more advantageous these mutations would be in the engineered organism.

The experimental observations in the current study are consistent with the structural models described in our previous work [23]. Mechanistic studies on DXS revealed that the catalytic reaction requires the formation of a ternary complex between GAP and C2α-lactylthiamine diphosphate (LThDP) intermediate, which is the predecarboxylation complex formed between pyruvate and ThDP [22, 26–28]. It has been observed that LThDP is a stable intermediate and its association with GAP facilitates the subsequent decarboxylation step to generate the enamine [22, 28]. It is likely that the mutations did not affect the interaction between LThDP and GAP as there was no change in the *K*_m_ for GAP. S1A Fig shows that the methyl group of Ala-147 is sticking out towards the open channel maintaining a distance of about 7.2 Å (S1B Fig) with the C2 atom of the thiazolium ring. On the other hand, the methyl group of Ala-352 is much closer (4.6 Å; S1B Fig) to the C2 atom of the thiazolium ring. Therefore, it is highly possible that in the WT enzyme, LThDP followed by the enamine adduct after decarboxylation would be oriented towards Ala-147 resulting in steric interaction between the bulky enamine adduct with the methyl group of Ala-147. Mutation of Ala-147 to Gly can be favorable for accommodating both the LThDP intermediate as well as the subsequent enamine adduct. This could explain the higher activity of A147G*Pt*DXS than the WT (Fig 2).

Loss of the methyl group of Ala-147 will also result in the loss of van der Waals interaction of the carbon backbone of ThDP (4.9 Å; S1B Fig). This loss of the hydrophobic interaction does not severely affect the binding of ThDP as there is another hydrophobic interaction (the backbone carbon of Gly-146, 4.0 Å; S1B Fig), which might be sufficient to keep the carbon backbone of ThDP in place. However, this might require a higher concentration of ThDP at the active site to compensate for the loss of this particular interaction with Ala-147. This could explain the higher *K*_m_ value of A147G*Pt*DXS for ThDP.

Mutation of Ala-352 to Gly possibly does not impair the binding of the terminal carbon of IDP. Apparently, this might be because of the presence of Leu-179, which can provide anchoring sites for the terminal carbons of IDP. On the other hand, this mutation may relieve the steric interaction between the methyl group of Ala-352 and the terminal carbon of IDP resulting in better binding of IDP and hence, more inhibition than the WT (Fig 3). This mutant was also found to have ~2.9 and ~2 times higher *K*_m_ values for ThDP and pyruvate respectively than those of the WT. Overall, the individual mutations of Ala-352 by Gly and Ala-147 to Gly have modulated the activity of *Pt*DXS and the mutated enzymes exhibited stronger inhibition compared to the WT.

In the case of the double mutant A147G/A352G*Pt*DXS, both the beginning and the end of the carbon chain of IDP suffer from inefficient binding, possibly because of the loss of key interactions of the termini of IDP with the methyl groups of Ala-147 and Ala-352. Consequently, this double mutant was also found to have reduced inhibition by IDP. This explains the higher *K*_*i*_ value of this mutant for IDP compared to the WT.

**Fig 4:**
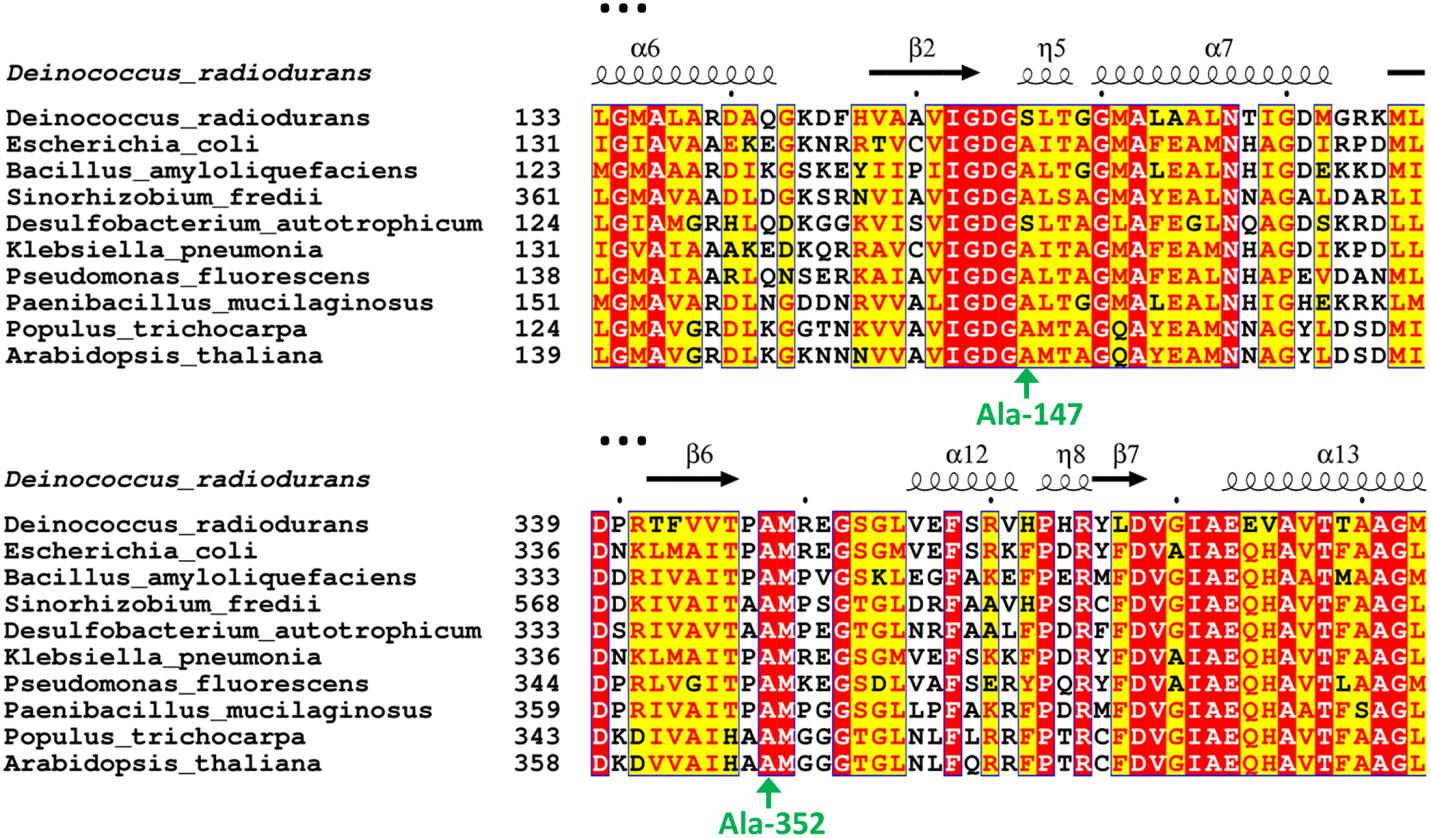
Excerpt from amino acid sequence alignment of DXS from a group of widely divergent bacteria and two plant species. The secondary structure elements of *D*. *radiodurans* enzyme are indicated on the top of the sequences. Conserved and similar residues are highlighted in red and yellow box respectively. The two Ala residues mutated in *Pt*DXS are indicated by green arrow. The sequence alignment has been generated using ESPript online server [30].

The residues Ala-147 and Ala-352, which have been mutated to glycine to probe their contribution in binding ThDP and IDP at the active site of the enzyme and also improving the activity of the engineered *Pt*DXS, are well conserved in bacteria and plants as observed from the amino acid sequence alignment of various DXS enzymes from a group of widely divergent bacteria and two plant species (Fig 4). It has been observed that Ala-352 of *Pt*DXS is completely conserved among all of these organisms. The absence of any natural mutation of this residue is evolutionarily critical and indicates the importance of this residue in the catalytic activity of the enzyme. This is also supported by this work as the mutation of Ala-352 to Gly makes the engineered enzyme less efficient. It has also been observed that Ala-147 of *Pt*DXS is highly conserved among all of these organisms except *Deinococcus radiodurans* and *Desulfobacterium autotrophicum*, which have Ser instead of Ala at the corresponding position of Ala-147 of *Pt*DXS. Despite the high degree of conservation at this position, it is intriguing to find that the activity of A147G*Pt*DXS has been modulated and can possibly be valuable in biotechnological applications. We can speculate that this variation could lead to altered kinetic parameters but similar enzyme efficiency. The absence of any natural mutation of Ala-147 of *Pt*DXS to Gly underscores the importance of its engineering to Gly to improve its activity for biotechnological purposes.

## Conclusion

The overall goal for this work was to test the effect of two key Ala residues in binding ThDP and IDP, and thus, on the activity of *Pt*DXS. Both the mutations exhibited some modifications in the enzyme kinetics and the binding affinity for both ThDP and the inhibitors. The mutation of Ala-147 to Gly has improved the activity of *Pt*DXS and slightly increased its inhibition by IDP. A352G*Pt*DXS exhibited inefficient binding with ThDP but stronger affinity for IDP compared to the WT. On the other hand, the double mutant exhibited slightly reduced activity and reduced inhibition by IDP compared to the WT. The results described here are analyzed in the context of the model illustrated in our previous work [23]. The experimental observations from this work corroborate with the predictions from the model. The binding pattern of both ThDP and the inhibitor IDP renders the engineering of *Pt*DXS for avoiding the feedback regulation really challenging [29]. This study shows that it is possible to modulate the feedback inhibition of the DXS enzyme for future biotechnological applications.

## Supporting Information

S1 Fig Cartoon view of the interactions of different residues of WT*Pt*DXS with IDP and their relevant distances from the carbon chain of IDP. (PDF)

S2 Fig A. Zoomed in surface view of the orientation of Ala-147 residue of WT*Pt*DXS and the thiazolium ring of ThDP in the enzyme active site. B. Cartoon view of the interactions of different residues of WT*Pt*DXS with ThDP and their relevant distances from the thiazolium ring and the carbon chain of ThDP.
(PDF)

S3 Fig SDS-PAGE of the different fractions from the Ni-NTA column purification of recombinant WT and the various mutants of *Pt*DXS. For WT panel, lane 1–3: elution fraction containing 50 mM imidazole; lane 4–5: elution fraction containing 100 mM imidazole; lane 6–7: elution fraction containing 150 mM imidazole. For A147G panel, lane 1: flow-through; lane 2–4: wash fraction containing 10 mM imidazole; lane 5–6: elution fraction containing 250 mM imidazole, lane 7: blank. For A352G panel and A147G/A352G panel, lane 1–2: elution fraction containing 50 mM imidazole; lane 3–4: elution fraction containing 100 mM imidazole; lane 5–6: elution fraction containing 150 mM imidazole; lane 7: elution fraction containing 200 mM imidazole. L: protein marker. The molecular weight of WT and all the mutant enzymes is ~73 kDa.
(PDF)
