# Correction: Engineering of Recombinant Poplar Deoxy-D-Xylulose-5-Phosphate Synthase (*Pt*DXS) by Site-Directed Mutagenesis Improves Its Activity

**DOI:** 10.1371/journal.pone.0165028

**Published:** 2016-10-13

**Authors:** Aparajita Banerjee, Alyssa L. Preiser, Thomas D. Sharkey

S1 Fig, S2 Fig and S3 Fig are the incorrect versions of these files. Please view the correct versions below.

## Supporting Information

S1 Fig A. Zoomed in surface view of the orientation of Ala-147 residue of WT*Pt*DXS and the thiazolium ring of ThDP in the enzyme active site. B. Cartoon view of the interactions of different residues of WT*Pt*DXS with ThDP and their relevant distances from the thiazolium ring and the carbon chain of ThDP.
(PDF)

S2 Fig SDS-PAGE of the different fractions from the Ni-NTA column purification of recombinant WT and the various mutants of *Pt*DXS. For WT panel, lane 1–3: elution fraction containing 50 mM imidazole; lane 4–5: elution fraction containing 100 mM imidazole; lane 6–7: elution fraction containing 150 mM imidazole. For A147G panel, lane 1: flow-through; lane 2–4: wash fraction containing 10 mM imidazole; lane 5–6: elution fraction containing 250 mM imidazole, lane 7: blank. For A352G panel and A147G/A352G panel, lane 1–2: elution fraction containing 50 mM imidazole; lane 3–4: elution fraction containing 100 mM imidazole; lane 5–6: elution fraction containing 150 mM imidazole; lane 7: elution fraction containing 200 mM imidazole. L: protein marker. The molecular weight of WT and all the mutant enzymes is ~73 kDa.
(PDF)

S3 Fig Michaelis-Menten plots for WT and different mutants of *Pt*DXS in presence of each of the substrates. Each data point represents mean, error bars represent S.E. (n = 3). Different symbols represent the experimental data points. The solid lines represent the fitted curves. Black, red, blue, and pink represent the activity of WT, A147G, A352G and A147G/A352G*Pt*DXS respectively.
(PDF)
