# Supplementary material for: Engineering of Recombinant Poplar Deoxy-D-Xylulose-5-Phosphate Synthase (PtDXS) by Site-Directed Mutagenesis Improves Its Activity
Source: PLoS One. 2016 Aug 22;11(8):e0161534. doi: 10.1371/journal.pone.0161534 (PMC4993486; doi:10.1371/journal.pone.0161534)
Supplement: S1 Fig — (PDF) [file pone.0161534.s001.pdf]

Engineering of Recombinant Poplar Deoxy-D-xylulose-5-phosphate Synthase (*Pt*DXS) by Site-directed Mutagenesis Improves Its Activity

Aparajita Banerjee[1], Thomas D. Sharkey[1*]

[1]Department of Biochemistry and Molecular Biology, Michigan State University, East Lansing, MI, USA

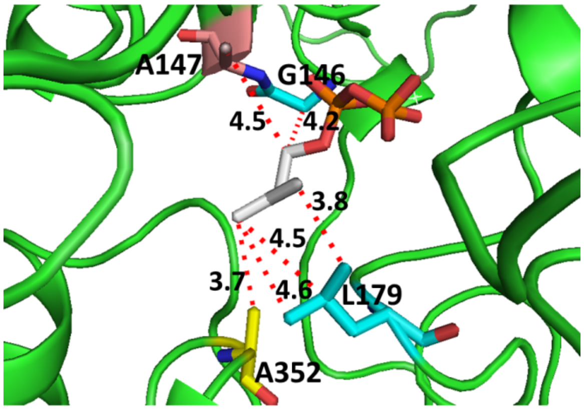

Fig S1. Cartoon view of the interactions of different residues of WT*Pt*DXS with IDP and their relevant distances from the carbon chain of IDP.
