# Supplementary material for: Correction: Engineering of Recombinant Poplar Deoxy-D-Xylulose-5-Phosphate Synthase (PtDXS) by Site-Directed Mutagenesis Improves Its Activity
Source: PLoS One. 2016 Oct 13;11(10):e0165028. doi: 10.1371/journal.pone.0165028 (PMC5063302; doi:10.1371/journal.pone.0165028)
Supplement: S1 Fig — A. Zoomed in surface view of the orientation of Ala-147 residue of WTPtDXS and the thiazolium ring of ThDP in the enzyme active site. B. Cartoon view of the interactions of different residues of WTPtDXS with ThDP and their relevant distances from the thiazolium ring and the carbon chain of ThDP. (PDF) [file pone.0165028.s001.pdf]

Engineering of Recombinant Poplar Deoxy-D-xylulose-5-phosphate Synthase (*Pt*DXS) by Site-directed Mutagenesis Improves Its Activity

Aparajita Banerjee[1], Alyssa Preiser[1], and Thomas D. Sharkey[1]

[1]Department of Biochemistry and Molecular Biology, Michigan State University, East Lansing, MI, USA

*Corresponding author

E-mail: [tsharkey@msu.edu]

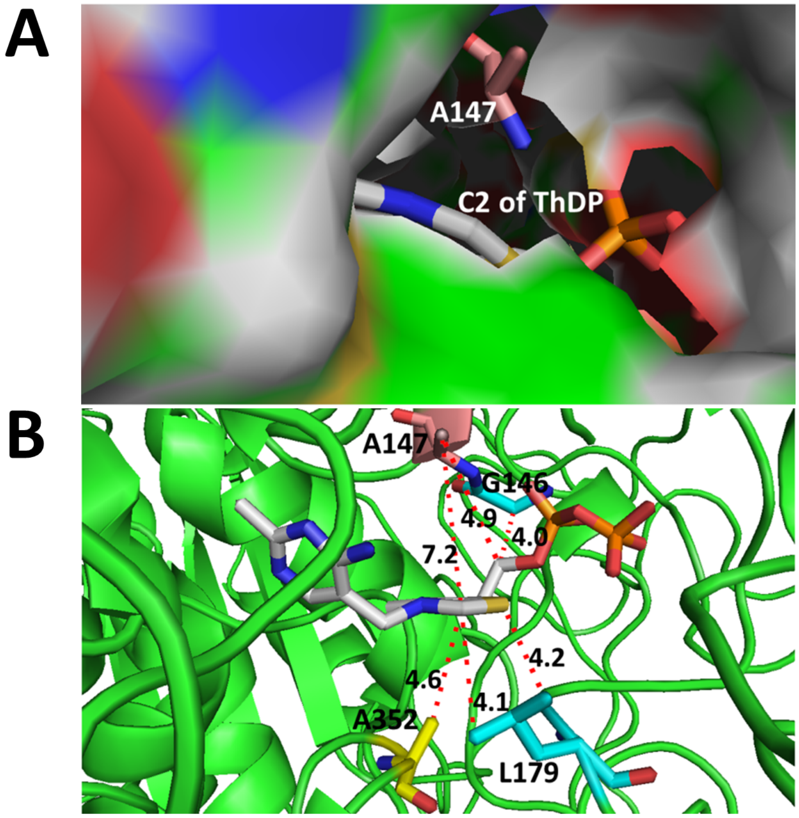

Fig S1. A. Zoomed in surface view of the orientation of Ala-147 residue of WT*Pt*DXS and the thiazolium ring of ThDP in the enzyme active site. B. Cartoon view of the interactions of different residues of WT*Pt*DXS with ThDP and their relevant distances from the thiazolium ring and the carbon chain of ThDP.
