# Supplementary material for: Correction: Engineering of Recombinant Poplar Deoxy-D-Xylulose-5-Phosphate Synthase (PtDXS) by Site-Directed Mutagenesis Improves Its Activity
Source: PLoS One. 2016 Oct 13;11(10):e0165028. doi: 10.1371/journal.pone.0165028 (PMC5063302; doi:10.1371/journal.pone.0165028)
Supplement: S2 Fig — For WT panel, lane 1–3: elution fraction containing 50 mM imidazole; lane 4–5: elution fraction containing 100 mM imidazole; lane 6–7: elution fraction containing 150 mM imidazole. For A147G panel, lane 1: flow-through; lane 2–4: wash fraction containing 10 mM imidazole; lane 5–6: elution fraction containing 250 mM imidazole, lane 7: blank. For A352G panel and A147G/A352G panel, lane 1–2: elution fraction containing 50 mM imidazole; lane 3–4: elution fraction containing 100 mM imidazole; lane 5–6: elution fraction containing 150 mM imidazole; lane 7: elution fraction containing 200 mM imidazole. L: protein marker. The molecular weight of WT and all the mutant enzymes is ~73 kDa. (PDF) [file pone.0165028.s002.pdf]

Engineering of Recombinant Poplar Deoxy-D-xylulose-5-phosphate Synthase (*Pt*DXS) by Site-directed Mutagenesis Improves Its Activity

Aparajita Banerjee[1], Alyssa Preiser[1], and Thomas D. Sharkey[1]

[1]Department of Biochemistry and Molecular Biology, Michigan State University, East Lansing, MI, USA

*Corresponding author

E-mail: [tsharkey@msu.edu]

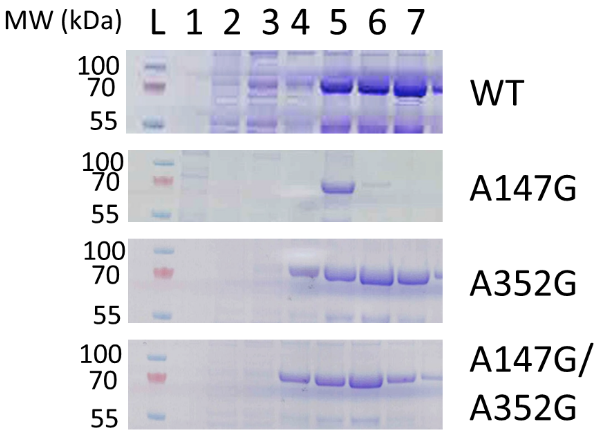

Fig S2. SDS-PAGE of the different fractions from the Ni-NTA column purification of recombinant WT and the various mutants of *Pt*DXS. For WT panel, lane 1-3: elution fraction containing 50 mM imidazole; lane 4-5: elution fraction containing 100 mM imidazole; lane 6-7: elution fraction containing 150 mM imidazole. For A147G panel, lane 1: flow-through; lane 2- 4: wash fraction containing 10 mM imidazole; lane 5-6: elution fraction containing 250 mM imidazole, lane 7: blank. For A352G panel and A147G/A352G panel, lane 1-2: elution fraction containing 50 mM imidazole; lane 3-4: elution fraction containing 100 mM imidazole; lane 5-6: elution fraction containing 150 mM imidazole; lane 7: elution fraction containing 200 mM imidazole. L: protein marker. The molecular weight of WT and all the mutant enzymes is ~73 kDa.
