# Supplementary material for: Correction: Engineering of Recombinant Poplar Deoxy-D-Xylulose-5-Phosphate Synthase (PtDXS) by Site-Directed Mutagenesis Improves Its Activity
Source: PLoS One. 2016 Oct 13;11(10):e0165028. doi: 10.1371/journal.pone.0165028 (PMC5063302; doi:10.1371/journal.pone.0165028)
Supplement: S3 Fig — Each data point represents mean, error bars represent S.E. (n = 3). Different symbols represent the experimental data points. The solid lines represent the fitted curves. Black, red, blue, and pink represent the activity of WT, A147G, A352G and A147G/A352GPtDXS respectively. (PDF) [file pone.0165028.s003.pdf]

Engineering of Recombinant Poplar Deoxy-D-xylulose-5-phosphate Synthase (*Pt*DXS) by Site-directed Mutagenesis Improves Its Activity

Aparajita Banerjee[1], Alyssa Preiser[1], and Thomas D. Sharkey[1]

[1]Department of Biochemistry and Molecular Biology, Michigan State University, East Lansing, MI, USA

*Corresponding author E-mail: [tsharkey@msu.edu]

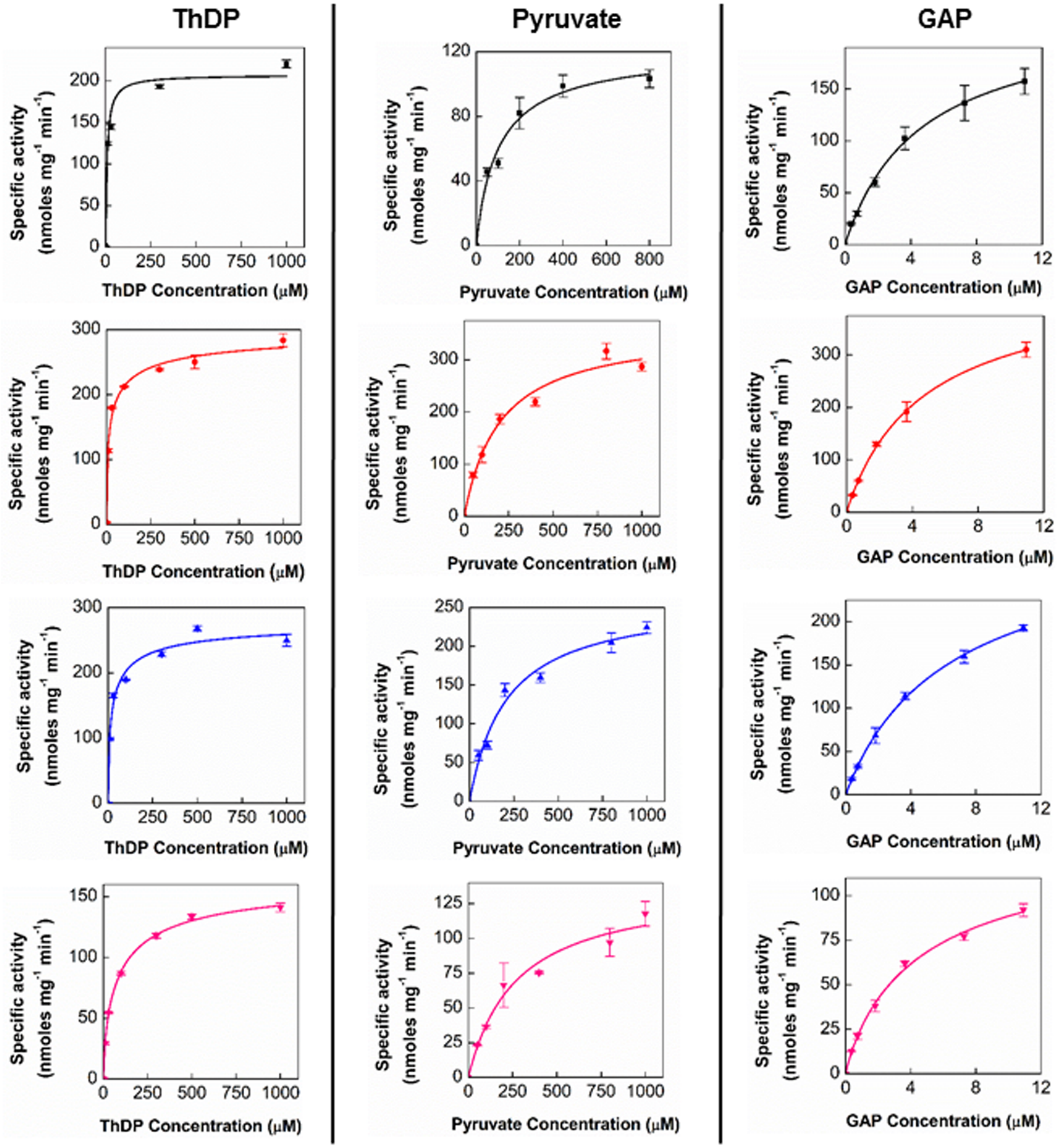

Fig S3. Michaelis-Menten plots for WT and different mutants of *Pt*DXS in presence of each of the substrates. Each data point represents mean, error bars represent S.E. (n = 3). Different symbols represent the experimental data points. The solid lines represent the fitted curves. Black, red, blue, and pink represent the activity of WT, A147G, A352G and A147G/A352G*Pt*DXS respectively.
